# Bonding and parent‐child quality of interaction in parents with eating disorder: A scoping review

**DOI:** 10.1002/erv.3144

**Published:** 2024-10-18

**Authors:** Maria Giulia Martini, See Heng Yim, Ivan Eisler, Nadia Micali, Ulrike Schmidt

**Affiliations:** ^1^ Institute of Psychiatry, Psychology and Neuroscience (IoPPN) King's College London London UK; ^2^ Children and Young People Eating Disorder Service Central and North West London NHS Foundation Trust London UK; ^3^ Great Ormond Street Institute of Child Health University College London UK; ^4^ Maudsley Centre for Child and Adolescent Eating Disorders (MCCAED) Maudsley Hospital London UK; ^5^ Department of Psychiatry University of Geneva Geneva Switzerland; ^6^ Mental Health Services in the Capital Region of Denmark Eating Disorders Research Unit Psychiatric Centre Ballerup Ballerup Denmark; ^7^ South London and Maudsley NHS Foundation Trust London UK

**Keywords:** children, eating disorders, intergenerational effect, parent‐child interaction, parents

## Abstract

**Objective:**

To summarise existing evidence on bonding and parent‐child quality of interaction in parents with eating disorder (ED).

**Methods:**

A scoping review was conducted. Seven databases (PsycInfo, Embase, Medline, Pubmed, OpenGrey, ProQuest and Google Scholar) were examined and studies exploring research into bonding and quality of interaction in parents with ED were included.

**Results:**

Seventeen quantitative studies were included in the review. Reviewed literature suggests that parents with ED tend to be more intrusive, may exhibit more negative expressed emotions and may be involved in higher levels of mealtime conflicts during mealtimes. Additionally, they may be less sensitive and may offer less structured in non‐feeding interactions. Children of parents with ED may have a more difficult temperament, may display greater internalisation and externalisation problems, may be less responsive to their parents and exhibit more behavioural difficulties.

**Conclusion:**

Overall, we found that parents with ED are more likely to have difficulties during interactions with children, compared with controls, both in feeding and non‐feeding contexts which might impact children's mental health. Proposals for future research are suggested to enhance our understanding of the intergenerational transmission of ED, holding the potential to pinpoint therapeutic and preventative targets for both parents with ED and their children.

## Key points

- This scoping review indicates that parents with eating disorder (ED) are more likely to have difficulties during interactions with children, compared with controls, both in feeding and non‐feeding contexts.
- These difficulties can potentially affect the socio‐emotional functioning and psychopathology of the children. Our findings also suggest that general and disorder‐specific aspects of parent‐child interaction will be central targets for preventative interventions.
- Future research should include fathers with ED and explore the influence of different ED diagnoses and their status (i.e. historical or current) on children's outcomes. More research is needed on protective factors that promote healthy adjustment and development in children of parents with ED.

## 1 INTRODUCTION

Eating disorders (ED) are common, with a lifetime prevalence of nearly 8.4% among women and 2.2% among men (Galmiche et al., 2019). Though they can affect individuals of all ages (Treasure et al., 2020), ED are typically diagnosed in adolescence and young adulthood and may persist chronically. Importantly, a significant number of adults with ED are also parents (Chapman et al., 2023).

Evidence indicates that children of parents with ED face heightened risks of experiencing both feeding and psychological difficulties (Martini et al., 2020), including the development of ED themselves (Bould et al., 2015). Underlying mechanisms are not entirely understood but are believed to encompass both genetic and environmental factors. Estimates of genetic heritability derived from family and twin studies indicate that genetic components play a significant role in anorexia nervosa (AN; 58%–74%), bulimia nervosa (BN; 59%–83%), binge‐eating disorder (BED; 41%–57%), and avoidant restrictive food intake disorder (ARFID; 79%; Bulik et al., 2022). Over the past decade, extensive research has been undertaken, aimed at enhancing our knowledge of genetics and neurobiology in ED (refer to Bulik et al., 2022 for a comprehensive review). Nonetheless, it is acknowledged that environmental factors likely contribute to the activation of genetic predispositions (Watson, O’Brien, & Sadeh‐Sharvit, 2018), highlighting the importance of investigating potential environmental mechanisms that may contribute to the development of detrimental outcomes in children.

Beyond genetic influences, ED could also be inherited from parents to children through the direct and indirect effects of ED symptomatology on overall parenting functioning (Patel et al., 2002). Mothers with current ED may struggle with emotional availability as their preoccupation with food, weight and body image may distract them from being fully present with their infant, which might potentially hinder the development of a secure emotional bond (Stitt & Reupert, 2014; Tuval‐Mashiach et al., 2013). Infants are also highly influenced by their caregivers, observing and internalising behaviours and attitudes. Parents with ED may inadvertently model unhealthy attitudes and behaviours related to food and body image, potentially influencing their child's perceptions and behaviours later in life (Chapman et al., 2021). Parents with ED also express having difficulties setting boundaries for their children (Stitt & Reupert, 2014) and describe themselves as being ‘inadequate’ in fulfiling the parental role (Franzen & Gerlinghoff, 1997; Tuval‐Mashiach et al., 2013). However, they are typically aware of these risks and they express significant concerns about the potential impact of their ED on their children's eating behaviours and body image, and report fears about their children possibly developing eating difficulties as well (Bryant‐Waugh et al., 2007; Chapman et al., 2023; Stitt & Reupert, 2014). Unsurprisingly, these fears seem to be even more salient in parents of daughters (Franzen & Gerlinghoff, 1997).

A robust body of evidence highlighted the significant impact of the quality of parent–infant verbal and nonverbal interaction on offspring's psychological well‐being and on the potential onset of psychopathologies in children (Cirulli et al., 2003). Numerous studies have demonstrated that the infant has an innate ability for mutual engagement in two‐way communication and demonstrates this early competence in interactional exchanges with their primary caregivers across various contexts (e.g., verbal interactions, feeding, and play) (Feldman, 2015; Markova & Legerstee, 2006). The quality of bonding and parent‐child interaction is influenced by a complex interplay of individual factors (e.g., genetic, neurobiological, temperamental), environmental influences, and relational dynamics. These interactions play a crucial role in shaping a child's ability to self‐regulate, which in turn can impact their emotional and behavioural development over time, potentially leading to maladaptive symptoms (Cerniglia, 2024).

Whilst bonding and parent‐infant quality of interaction have been examined for other psychiatric disorders (Badr et al., 2018; Campbell et al., 2004; Nath et al., 2019), there have been limited literature reviews to synthesise bonding and parent‐infant quality of relationship in parents with ED (Chapman et al., 2021; Martini et al., 2020; Watson et al., 2018) and no scoping review has been conducted so far. It was therefore deemed to be more appropriate to carry out a scoping review rather than a systematic review of this emerging literature (Peters et al., 2020) as we aimed for a broader scope incorporating grey literature, non‐clinical populations and ARFID population, which have been overlooked in previous studies. Additionally, we aimed to condense existing evidence on the impact of maternal ED on bonding and parent‐child interaction, whilst also striving to identify and analyse knowledge gaps (Peters et al., 2020).

Understanding the full impact of ED on bonding and quality of mother‐infant interaction for ED and in relation to children at different developmental stages could inform further development of interventions. Such interventions have the potential to not only offer much‐needed support for parents with ED, and may also mitigate the intergenerational transmission of ED.

The primary objective of this study is to provide an overview of the evidence on bonding and mother‐infant quality of interaction in parents with ED. Additionally, we aim to present an overview of the different outcomes linked to bonding and the quality of parent‐infant interaction in parents with ED. This review could help identify potential targets for future research.

## 2 METHODS

A scoping review methodology outlined by Peters et al. (2015), was employed to examine the existing research on bonding and quality of interaction in parents with ED. This approach was deemed the most suitable considering the heterogeneity of existing research and the broad aims of this review. Current guidelines for scoping reviews (Peters et al., 2020) and Preferred Reporting Items for Systematic Reviews and Meta‐Analyses (PRISMA) extension for scoping reviews guidance (Tricco et al., 2018) were adhered to during the review process. Before conducting the search, a protocol was developed but has not been published.

### 2.1 Search strategy

PsycInfo, Embase, Medline and Pubmed databases were searched on the 25th of September 2023 and a further search was conducted on the 14th of April 2024. Search terms are outlined in Supporting Information S1: Appendix 1. Additionally, hand‐searches of articles, reference lists and the internet were executed. Whilst systematic searches were performed in the above databases, OpenGrey, ProQuest and Google Scholar were examined manually using different combinations of the search terms and keywords to randomly inspect if any additional publications were identified. Inclusion criteria for publication were as follows: (1) exposure, that is, (mothers and fathers) diagnosed with any ED and disordered eating including AN, BN, BED, ED not otherwise specified (EDNOS)/other specified feeding and ED (OSFED), or ARFID, based on the Diagnostic and Statistical Manual IV or 5 (American Psychiatric Association, 2000, 2013), either current or historical, (2) the outcome was a measure of either child bonding or mother‐child quality of interaction, evaluated from infancy through 12 years of age, (3) research published in English language (4) primary studies. Studies were excluded if their objectives focused on examining offspring eating pathology rather than maternal ED.

### 2.2 Selection process

Following the initial search, duplicates were removed and MGM and SHY independently reviewed the abstracts and titles of the remaining studies. The full‐text of the remaining citations was screened for eligibility by both authors, and a consensus was reached on which paper to include in this synthesis.

### 2.3 Data charting, extraction and analysis

A data charting table was developed by MGM and reviewed by US. The development process drew upon the JBI Manual for Evidence Synthesis and other guidelines and reviews on similar topics (Hunter et al., 2022).

All included articles were charted based on study methodology characteristics and results regarding bonding and mother‐child interactions (Table 1). Study design characteristics encompassed sample size, the publication year, age of participants and diagnosis. Results comprised exposures and outcome measures, differences in bonding and mother‐child interactions and whether these differences were associated with differential outcomes for children of parents with ED as opposed to children of control parents. A narrative synthesis approach was employed to identify themes and patterns across the studies with reference to Popay et al. (2006). The synthesis stages involved identifying conceptual categories based on the focus of the review and assessing the strength of the evidence. It is important to note that a scoping review does not aim to critically appraise evidence but to provide an overview of an emerging field (Peters et al., 2020). Within the narrative synthesis framework (Popay et al., 2006), critical reflection was used to evaluate the methodology, quality, validity and generalisability of the findings, as well as any discrepancies to evaluate the robustness of the evidence. Inconsistencies were addressed through discussion between MGM and SHY.

**TABLE 1:** Studies exploring the impact of parental ED on bonding and mother‐infant quality of interaction.

Authors; year order	Study, design, clinical/non‐clinical sample	Participants, *N*, maternal age, diagnoses, recruitment, age of child (range)	Measures (ED exposure and outcome)	Themes and findings
1. Stein et al., 1994	Case‐control	Total *n* = 58 mothers.	Exposure: Maternal ED (clinical interview through eating disorder examination ‐EDE)	Negative expressed emotion more frequent among the case mothers compared to HC during mealtimes but not play. The precursors were related to three main issues: (a) The mother's perception of mess; (b) issues of control; and (c) food refusal
An observational study of mothers with eating disorders and their infants.		ED cases *N* = 34; EDNOS *N* = 12, BN *N* = 6 & subthreshold *N* = 16; mean age 28.3		ED mothers were less facilitating during both mealtimes and play, had significantly more conflict with infants, but there was no difference in frequency or quality of mother‐infant physical contact.
Both mealtime and non food related (play)	Clinical sample	Healthy control (HC) *N* = 24; mean age 29.0	Outcome: Infant development: Bayley scales feeding and growth: Tanner and Whitehouse's specification	Case infants were rated as less happy than the controls during both mealtime and play
		Children age 12–14 months, mean 12.6 (SD 0.6)		
2. Stein et al., 1999	Case‐control	Total *N* = 58	Exposure: Maternal ED (clinical interview DSM III‐R)	More meal‐time conflicts in the index group when compared to HC.
Conflict between mothers with eating disorders and their infants during mealtimes.	Clinical sample	Cases *N* = 34, mean age = 28.3 (range 21–42)	Outcome: Five‐point conflict/Harmony scale	Conflict less likely in the index group when mothers acknowledged infant's cues and were able to put aside their concerns.
Mealtime		HC *N* = 24, mean age = 29 (range 22–39)		The most frequent antecedent to conflict was the mother's concern about the manner of eating; disagreement over who fed the infant and food refusal.
		Children age 12–14 months, mean 12.6 (SD 0.6)		ED mothers acknowledged infant's signals in a third of cases compared to over a half in HC.
3. Waugh and Bulik (1999)	Case‐control	Total *N* = 20 mothers	Exposure: Maternal ED (clinical interview DSM III‐R)	No differences observed in childhood temperament or mothers' satisfaction with children's appearance.
Offspring of women with eating disorders.	Clinical sample	*N* = 10, mean age 30.1 (SD 3.1)	Outcome: Toddler temperament scale (TTS); mealtime observation schedule (MOS); food diary	Mothers with current or past ED made significantly fewer positive comments about food and eating during meal times.
Mealtime		Past AN = 6, past BN = 7 HC *N* = 10, mean age 30.8 (SD 3.6)		
		Children age 12–48 months, mean 30.8 (SD 13.2)		
4. Stein et al., 2001	Case‐control	Total *N* = 134 mothers	Exposure: Maternal ED (clinical interview through eating disorder examination ‐EDE)	Mothers with ED used more verbal control, especially strong control (i.e. commands, prohibits, forbids, cautions or corrects) than HC.
Influence of psychiatric disorder on the controlling behaviour of mothers with 1‐year‐old infants: A study of women with maternal eating disorder, postnatal depression and a healthy comparison group.	Clinical sample	ED mothers *N* = 34; mean age 28.3 (SD 21–42)	Outcome: Mother and child were videotaped during mealtime and play	No differences found between the groups on gentle verbal control and physical contact. Maternal dietary restraint was the one feature of ED psychopathology associated with the use of verbal control.
Both mealtime and non‐food related		Mothers with postnatal depression *N* = 39; mean age 28.0 (SD 18–40)		Marital criticism was also associated with the extent of verbal controlling behaviour.
		HC *N* = 61; mean age 28.4 (SD 20–41)		
		Children age 12–14 months, mean age 12.6 (SD 0.6)		
5. Park et al., 2003	Case‐control	Total *N* = 57 Mothers with ED *n* = 33, mean age 32 (range 26–46)	Exposure: Maternal ED (clinical interview through eating disorder examination ‐EDE); schedule for affective disorders and schizophrenia or SADS	Positive representations of the mother expressed as feeding, eating or body shape themes were more frequent in the ED group.
Children's representation of family mealtime in the context of maternal eating disorders.	Clinical sample	Healthy controls *n* = 24, mean age 33 (range 26–43)	Outcome: Mother and child were videotaped enacting a family mealtime in pretend play.	There were no other significant group differences in representations. Current maternal eating psychopathology was the principal predictor of these positive maternal representations.
Mealtime		Children age 12–14 months (T1) (mean age not specified); 4 years and 9 months (T2), mean age 5.1 SD 0.14		
6. Blissett et al., 2006	Uncontrolled	Total *N* = 94 co‐habiting mother–father dyads, mean age 36.4 years, (SD = 4.9)	Exposure: EDI‐2	Mothers reported greater perceived feeding responsibility and greater monitoring of their children's food intake than fathers. Bulimia scores were correlated with controlling feeding practices in mothers of girls but not boys.
Maternal and paternal controlling feeding practices with male and female children.	Non‐clinical sample	Children age 12–62 months, mean age 37.7 months, SD 12.7	Outcome: The child feeding questionnaire (CFQ)	Fathers' body dissatisfaction was correlated with monitoring of sons' but not daughters' food intake.
Mealtime				
7. Haycraft and Blissett (2010)	Uncontrolled	Total *n* = 105 mothers, non‐clinical sample, mean age of 35 years (SD 4.65)	Exposure: Eating disorder Inventory‐2	Higher levels of ED symptoms were associated with more authoritarian and permissive parenting styles.
Eating disorder symptoms and parenting styles	Non‐clinical sample	Children age 27–51 months (mean 39 months (SD 11.67))	Outcome: Parenting styles and dimensions questionnaire (PSDQ)	Authoritative parenting was not significantly related to ED symptoms.
Non‐food related (Parenting style)				
8. Blissett and Haycraft (2011)	Uncontrolled	Total *n* = 23 mothers‐fathers pairs, mean age of the mothers was 36 years (S.D. = 4.39, range 29–46) and fathers' mean age was 37 years (S.D. = 4.54, range 31–49).	Exposure: Eating disorder Inventory‐2	Parental reports of ED symptoms were related to observations of greater verbal pressurising by both parents, maternal restriction of children's food intake and use of incentives to eat, more mouthfuls of food eaten by the child, and less food refusal.
Parental eating disorder symptoms and observations of mealtime interactions with children	Non‐clinical sample	Children age 18–67 months (mean 37, SD 13.96)	Outcome: Mealtime observations coding through family mealtime coding system	
Mealtime				
9. Haycraft et al., 2015	Uncontrolled	Total *N* = 170 mothers, mean age was 39 years (SD 5.08, range 24–54 years)	Exposure: EDE‐Q	Mothers' eating psychopathology and exercise beliefs predicted activity parenting practices with their sons and daughters, but different predictors were seen for mothers of daughters versus sons. In particular,
Activity‐related parenting practices: development of the Parenting Related to Activity Measure (PRAM) and links with mothers' eating psychopathology and compulsive exercise beliefs.	Non‐clinical sample	Children age 4.5–9 years old (mean 7, SD 1.21)	Outcome: Compulsive exercise test (CET‐exercise); parenting related to activity measure	High CET‐exercise rigidity scores predicted high PRAM‐activity regulation scores but lower levels of EDEQ‐restraint in mothers also significantly predicted higher maternal control of their son's active behaviours.
Non‐food related (Interactions about physical exercise)				Higher child BMI SDS predicted mothers' concern about overweight in their daughters.
Study 2				
10. De Barse et al., 2015	Cohort study	Total *n* = 4851 Generation R study Cases	Exposure: Maternal ED (self‐report questionnaire)	Mothers with a history of ED used less pressure to eat than mothers without.
Does maternal history of eating disorders predict mothers' feeding practices and preschoolers' emotional eating?	Clinical	*N* = 415, mean age = 30.8 (SD 5.0) History of AN = 121, history of BN = 189, history of AN + BN = 105	Outcome: Child feeding questionnaire (CFQ); child eating behaviour questionnaire (CEBQ); Children's body mass index	Mothers with a history of AN were likely to use low levels of pressure to eat.
Mealtime		Healthy controls *N* = 4,436, mean age = 30.8 (SD 4.8)		Children of mothers with an ED had higher levels of emotional overeating than controls—this was strongest with mothers with a history of AN
		Children age 4 years old (not specified mean and range)		Maternal history of BN was not related to mothers' feeding practices or children's emotional eating.
11. Saltzman et al., 2016a	Case‐control	Total *N* = 441 parent‐child pairs, age not specified	Exposure: Eating disorder diagnostic scale (EDDS)	Parent's binge eating was correlated with distress responses, restriction for health, and restriction for weight control.
Parent binge eating and restrictive feeding practices: Indirect effects of parent's responses to child's negative emotion.		Non‐clinical sample	Outcome: Comprehensive feeding practices questionnaire (CFPQ)	Controlling for confounders, binge eating was associated with restriction for weight control, and restriction for health.
Mealtime		Children age not specified (pre‐school children)		
12. Saltzman et al., 2016b	Case‐control	Total *N* = 260	Exposure: Frequency of BED behaviours through self‐report questionnaire eating disorder diagnostic scale (EDDS)	Maternal BED predicted use of more nonresponsive feeding practices (e.g. Emotion regulation, restriction for health, pressure to eat, and food as reward).
Eating, feeding, and feeling: emotional responsiveness mediates longitudinal associations between maternal binge eating, feeding practices, and child weight.	Non‐clinical sample	Cases *N* = 36 mothers with BED, mean age not specified	Exposure: Children's negative emotions scale; comprehensive feeding practices questionnaire;	Maternal BED was associated with greater use of distress responses, which indirectly predicted higher child BMI percentile through food as reward feeding practices.
Mealtime		HC *N* = 224, mean age not specified	Child BMI	
		Children age 30–44 months; 37 months (SD 6.9) (wave 1); 49–65 months 57 months (SD 8.3) (wave 2)		
13. Cimino et al., 2016	Case‐control	Total *N* = 408 parents	Exposure: Maternal ED (clinical interview through DSM‐5 criteria)	Groups with one or both parents diagnosed with BED showed higher scores on the SVIA and on the CBCL internalising and externalising scales, indicating poorer adult–child feeding interactions and higher emotional–behavioural difficulties.
Mothers and Fathers with Binge Eating Disorder and Their 18–36 Months Old Children: A Longitudinal Study on Parent‐Infant Interactions and Offspring's Emotional‐Behavioural Profiles.	Clinical	Cases both parents with BED *N* = 102, Children = 51 only mothers with BED *N* = 104, Children = 52 only fathers with BED *N* = 100, Children = 50	Outcome: Scale for the assessment of feeding interactions (SVIA)/Child behaviour Check‐List (CBCL)	A direct influence of parental psychiatric diagnosis on the quality of mother–infant and father–infant interactions was also found, both at 18 (T1) and 36 months of the child (T2).
Mealtime		Healthy controls *N* = 102 parents, children *N* = 51		
		Children age 18 months (T1) and 36 months (T2)		
14. Sadeh‐Sharvit et al., 2016	Case‐control	Total *n* = not specified	Exposure: Self‐report questionnaire (EDI‐2)	Mothers with ED were less sensitive to their children, tried to control their children's behaviours more, and were less happy during mother–child interactions.
The interactions of mothers with eating disorders with their toddlers: identifying broader risk factors.	Clinical sample	Cases *N* = 29; AN = 14; BN = 13; EDNOS = 2, mean age = 31 (4.20)	Outcome: CBCL/2–3; video recording of mother/child interaction	The children in the maternal ED group were rated as less responsive to their mothers and their mothers also reported more behavioural problems than those in the control group.
Non‐food related (playtime)		HC *n* = not specified, mean age, 33.1 (SD 4.64)		
		Children age 18–42 months		
15. Cimino et al., 2018	Case‐control	*N* = 200; mean age, 33.1 (4.64) Total *N* = 150 families	Exposure: Maternal ED, clinical interview (SCID‐I)	Children with both parents with BED showed the highest affective, anxiety, oppositional/defiant, and autism spectrum problems, but no influence of paternal diagnosis was found on the offspring's psychopathology.
Impact of parental BED: exploring children's emotional/behavioural problems and the quality of parent‐child interactions	Clinical sample	Cases F+/M + *N* = 50 M−/F + *N* = 50 F+/M− *N* = 50 HC = 50	Outcome: Italian adaptation of feeding scale (SVIA)/CBCL	Maternal BED had an influence on children's affective and autism spectrum problems,
Mealtime		Children age 18 months (T1) and 36 months (T2)		Diagnosis of BED in both parents had an effect on infants' affective problems
				Paternal BED had an effect on oppositional/defiant problems through the quality of father–infant interactions,
				Maternal BED had an effect on the offspring's affective and anxiety problems through the mediation of mother–infant interactions
16. Martini et al., 2022	Case‐control	Total *N* = 62	Exposure: Maternal ED, clinical interview (SCID‐I)	No differences were found between early mother‐infant interaction and bonding in mothers with ED in comparison to HC.
Effect of maternal eating disorders on mother‐infant quality of interaction, bonding and child temperament: A longitudinal study.	Clinical sample	ED *N* = 36; (current ED = 16; past ED = 20), mean age 31.92 (SD 5.70)	Outcome: CARE‐INDEX; mother‐to‐infant bonding scale (MIBS); early childhood behaviour questionnaire (ECBQ)	High levels of maternal ED psychopathology were correlated with high anxiety levels, higher negative affectivity and lower extroversion in children of ED mothers both at 1 and 2 years.
Non‐food related (playtime)		HC *N* = 26, mean age 33.92 (SD 4.04)		Comorbid anxiety and depression in mothers with ED were associated with worse mother‐child bonding.
		Children age 8 weeks (T1), 1 year (T2), 2 years (T3) (range and mean not specified)		
17. Loth et al., 2024	Cohort	Total *N* = 1306 children, mean age 7.0 (SD 1.5) and 1130 parents; mean age 35.7 (SD 7.8)	Exposure: Self‐reported questionnaire regarding parent engagement in restrictive or compensatory disor‐ dreed eating behaviours or binge eating	Parents engaging in restrictive disordered eating behaviours and binge eating reported significantly higher levels of coercive food parenting practices, including pressure‐to‐eat, restriction, threats and bribes, and using food to control negative emotions.
Associations between parental engagement in disordered eating behaviours and use of specific food parentingpractices within a racially, ethnically, and socioeconomically diverse sample	Non‐clinical sample	Disordered eating behaviours: *N* = 544 engaged in restrictive behaviours; *N* = 146 engaged in compensatory behaviours; *N* = 97 engaged in binge eating behaviours	Outcome: Use of specific food parenting practices	Parental engagement in restrictive disordered eating behaviours was also associated with significantly higher use of food rules and limits.
Food parenting practices				

Abbreviations: AN, anorexia nervosa; BED, binge eating disorder; BN, bulimia nervosa; ED, eating disorder cases; HC, healthy controls; SD, standard deviation.

## 3 RESULTS

**FIGURE 1:**
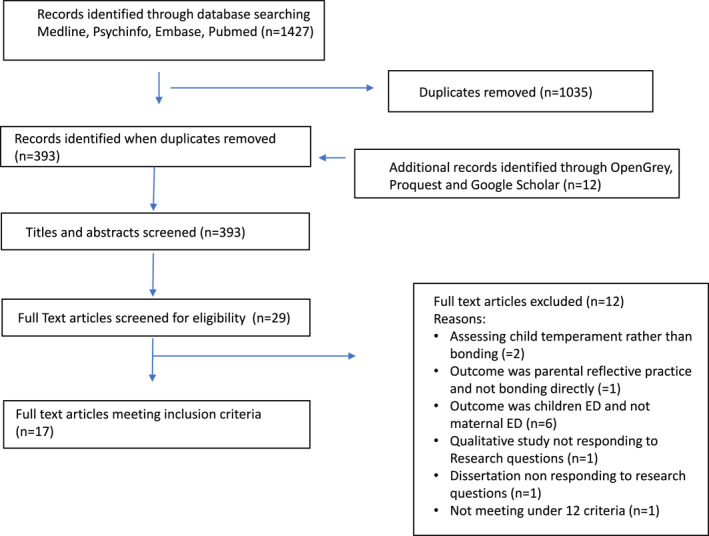
Review search and selection process. *Source*: (PRISMA, Moher et al., 2009).

The literature search initially yielded three‐hundred‐and‐93 papers. After duplicates were deleted and screening performed according to eligibility criteria, a total of 17 studies met the inclusion criteria for the review (refer to Figure 1 for the PRISMA flowchart). Eleven out of 17 were case‐control studies, two had a cohort design and four were uncontrolled. Six studies included both maternal and paternal ED (Blissett & Haycraft, 2011; Blissett, Meyer, and Haycraft, 2006; Cimino et al., 2016, 2018; Loth et al., 2024; Saltzman, Liechty, et al., 2016).

## 4 OVERVIEW OF BONDING AND MOTHER‐INFANT QUALITY OF INTERACTION IN PARENTS WITH ED

Most studies explored interactions during mealtime (Waugh & Bulik, 1999; Park et al., 2003; Stein, 1999; Blissett & Haycraft, 2011; de Barse et al., 2015; Saltzman, Pineros‐Leano et al., 2016; Cimino et al., 2016, 2018; Saltzman, Liechty, et al., 2016; Blissett, Meyer, and Haycraft, 2006; Loth et al., 2024), two studies investigated both mealtimes and non‐food related interactions (Stein et al., 1994, 2001) and three studies examined non‐food related interactions between parent and child (Haycraft & Blissett, 2010; Haycraft et al., 2015; Martini et al., 2022).

### 4.1 Interactions during mealtime

Themes examined in feeding context were mealtime expressed emotions (Stein et al., 1994), intrusiveness (Stein et al., 1994), amount of conflicts (Stein et al., 1994, 1999), verbal controlling statements and controlling/non‐responsive/coercive feeding practices (Blissett & Haycraft, 2011; Jacqueline Blissett, Meyer, and Haycraft, 2006; Saltzman, Liechty, et al., 2016; Saltzman, Pineros‐Leano, et al., 2016; Stein et al., 2001; Waugh & Bulik, 1999). One study reported on children's play representations of family mealtimes (Park et al., 2003).

In the first of a series of observational studies conducted by Stein and colleagues, findings revealed that ED mothers displayed higher levels of intrusiveness, experienced higher levels of conflicts during mealtime and demonstrated more frequently negative expressed emotion during mealtimes (but not during play) compared to control mothers (Stein et al., 1994). The precursors of negative expressed emotion were related to the mother's perceptions of mess, issues of control and food refusal. In a subsequent study carried out by Stein et al. (1999), further exploration of mealtime conflict in mother‐child dyads revealed higher levels of conflict in the index group compared to controls. Remarkably, conflicts were less likely to occur when mothers recognised and responded to the infant's cues and were able to set aside their concerns (Stein, 1999). In a subsequent study carried out by Stein et al. (2001), mothers with ED expressed a higher percentage of controlling statements compared to mothers with postnatal depression and healthy controls. This difference became non‐significant when adjusted for covariates. Dietary restraint and marital criticism emerged as significant predictors of the percentage of verbal controlling to total statements (Stein et al., 2001).

Similar findings were described by Waugh and Bulik (1999), where mothers with both current and past ED were found to make considerably fewer positive remarks regarding food and eating during mealtimes. However, no significant differences were observed between groups for other maternal mealtime behaviours including maternal praise, physical contact, instructions, behaviours related to presenting and removing food, social attention, and non‐interaction behaviours.

In the study conducted by Park et al. (2003), differences in children's perceptions of family mealtimes were explored using doll house figures, furniture, cutlery, and toy food with children of mothers with ED and control mothers with a child aged approximately 5 years. When asked to engage in pretend family mealtime using the toys, the children of mothers with ED depicted significantly more positive representations of their mothers in relation to themes related to eating and body shape than controls. Current maternal ED psychopathology emerged as the main predictor of these positive maternal depictions. On the other hand, criticism within marital relationships was associated with negative maternal depictions (Park et al., 2003).

Only few studies included both maternal and paternal ED in their sample. In a study aimed at comparing both maternal and paternal feeding practices and examining the impact of parent and child gender on the relationship between controlling feeding practices and parental unhealthy eating attitudes, Blissett and colleagues (2006) found that mothers expressed a greater sense of feeding responsibility and increased monitoring of their offspring's dietary intake compared to fathers. Additionally, BN scores were associated with controlling feeding practices in mothers of daughters but not sons, whereas fathers' body dissatisfaction correlated with monitoring of boys' but not girls' dietary intake (Blissett, Meyer, and Haycraft, 2006). In a later study conducted by the same authors, ED symptoms were associated with observed instances of increased verbal pressurising by both parents, maternal restriction of offspring's dietary intake and use of incentives to encourage eating. Additionally, children consumed more mouthfuls of food, whilst exhibiting less food refusal (Blissett & Haycraft, 2011). Similarly, in a recent prospective cohort study aimed at exploring the use of specific food parenting practices in parents engaging in disordered eating behaviours (i.e. restriction, compensatory and binge eating behaviours), the authors found a significant association between parental engagement in any type of disordered eating behaviours and increased use of coercive food parenting practices (i.e. pressure‐to‐eat, restriction, threats and bribes, and using food to control negative emotions; Loth et al., 2024).

Differences between ED diagnoses and their effects on interaction with children during mealtimes are noteworthy. Saltzman and colleagues found that maternal BED was predictive of increased use of non‐responsive feeding practices including using food for emotion regulation, imposing food restrictions for health reasons, exerting pressure to eat and offering food as a reward. This association was mediated indirectly, through intensified reactions to negative emotions displayed by children. Maternal BED was significantly related to increased distress responses, which indirectly predicted higher body mass index (BMI) percentile in offspring via the use of food‐as‐reward feeding methods (Saltzman et al., 2016). Furthermore, parental BED was correlated with distress responses, food restriction for health reasons, and food restriction for weight control. When accounting for confounding factors, binge eating was associated with restriction for weight control and health (Saltzman, Liechty, et al., 2016). Finally, mothers with past AN were more inclined to employ less pressurising feeding strategies in comparison to healthy controls (de Barse et al., 2015).

### 4.2 Interactions in non‐mealtime context

Themes examined in non‐feeding context were expressed emotions, controlling verbalisation, frequency of parental vocalisations and physical contact (Martini et al., 2022; Stein et al., 1994, 2001), emotional availability, sensitivity and non‐hostility (Martini et al., 2022; Sadeh‐Sharvit et al., 2016), intrusiveness, structure and facilitation (Sadeh‐Sharvit et al., 2016; Stein et al., 1994), parenting style (Haycraft & Blissett, 2010) and interactions about physical exercise (Haycraft et al., 2015).

In the study cited above by Stein and colleagues, it was observed that mothers with ED voiced a higher proportion of controlling statements during play compared to non‐ED mothers. In a subsequent paper where mothers with ED were compared to mothers with postnatal depression as well as healthy controls, the proportion of controlling statements made was found to be predicted by maternally‐reported marital criticism (Stein et al., 2001). Additionally, mothers with ED displayed higher levels of strong verbal control statements such as commands and cautions during play compared to control groups. However, they did not differ in the use of gentle control statements such as suggestions and prompts from either comparison group (Stein et al., 2001).

Sadeh‐Sharvit et al. (2016) and Stein et al. (1994) converged in finding that mothers with ED exhibited higher levels of intrusiveness during play with their children compared to controls. Additionally, these mothers were reported to be more ‘hostile’ and offered less structured guidance during play compared to controls (Sadeh‐Sharvit et al., 2016). Similar findings were reported by Stein et al. (1994), where it was found that mothers with postnatal ED were less facilitating, meaning they assisted their child less during play, compared to healthy controls (Stein et al., 1994). No differences were noted by Stein and colleagues in the frequency of parental vocalisations and physical contact between mother‐child dyads with ED and controls.

Haycraft and Blissett (2010) examined the relationship between ED symptoms and parenting style in a non‐clinical population (Haycraft & Blissett, 2010). The authors found an association between increased severity of ED symptoms and both permissive and authoritarian parenting styles, which are considered less adaptive styles of parenting. However, caution is warranted in interpreting these findings as the data in this study were cross‐sectional and additional variables such as rigidity of thinking and comorbid psychopathology (i.e. OCD traits etc), which may have an impact on this relationship, were not considered.

In a later study conducted by the same authors, maternal attitudes toward physical exercise, measures of ED psychopathology, and excessive exercise were examined to determine predictors of parenting behaviours related to physical activity. They found that maternal eating psychopathology and attitude toward physical exercise influenced parenting practices related to physical activity with children of both genders. However, distinct predictors of parenting practices related to physical activity were observed in mothers of sons compared to mothers of daughters. Child BMI emerged as an important predictor of maternal activity‐related parenting. Particularly, it predicted increased concern about children of both genders being overweight, greater monitoring of physical activity in boys and increased pressure to engage in physical exercise in girls (Haycraft et al., 2015).

## 5 BONDING, MOTHER‐INFANT QUALITY OF INTERACTION AND RELATED OUTCOME IN CHILDREN OF MOTHERS WITH ED

Five studies focused on bonding/parent‐infant quality of interaction and associated outcomes in children whose mothers had ED (Cimino et al., 2016, 2018; de Barse et al., 2015; Sadeh‐Sharvit, Levy‐Shiff, Arnow, et al., 2016; Martini et al., 2022). A small case‐control study (Sadeh‐Sharvit, Levy‐Shiff, Arnow, et al., 2016), found that mothers with ED were less sensitive to their toddlers (18–42 months), attempted to exert more control over their children's behaviours, and exhibited less happiness during interactions with their children. The offspring within the maternal ED group were observed to be less responsive to their caregiver and their mothers reported a higher incidence of behavioural difficulties compared to controls.

Two longitudinal studies explored the emotional and behavioural profile of children (aged 18 months at T1 and 36 months at T2) and parent‐child interaction during feeding in the context of a parental diagnosis of BED. The authors discovered that offspring having one or both parents with a BED diagnosis exhibited greater scores on scales measuring both internalising and externalising behaviours, as well as elevated levels of behavioural and emotional problems (Cimino et al., 2016). Moreover, maternal BED was found to be linked to emotional difficulties in children, and the presence of a BED diagnosis in both parents impacted on the emotional difficulties experienced by the infants. Whilst paternal BED was associated with oppositional‐defiant difficulties, maternal BED had an impact on the offspring's affective and anxiety problems. Both associations were mediated through parent‐infant quality of interaction (Cimino et al., 2018).

In a case‐control study conducted by our research group, it was found that increased severity of maternal ED psychopathology was associated with heightened levels of anxiety, increased negative affective state, and decreased extraversion in offspring of mothers with an ED diagnosis both at ages 1 and 2. Additionally, greater severity of ED psychopathology was associated with reduced levels of effortful control at 1 year. However, no differences were identified in early mother‐infant interaction and bonding between mothers with ED compared to healthy controls (Martini et al., 2022).

In a large population‐based cohort study, children of mothers with AN exhibited increased level of emotional overeating. Additionally, mothers with AN were found to employ fewer pressuring feeding strategies. No significant differences were observed between offspring of mothers with BN and healthy controls (de Barse et al., 2015).

## 6 DISCUSSION

The present scoping review extends our previous understanding of the impact of parental ED on bonding and quality of parent‐infant interaction and related outcomes in offspring of parents with ED. Overall, our findings indicated that parents with ED are more likely to have difficulties during interactions with children, compared with controls, both in feeding and non‐feeding contexts with potential impact on children's mental health.

Generally, the papers varied in quality and the majority of the studies examined interactions in relation to feeding and mealtimes whilst only a handful of studies examined infant‐parent interactions and related outcomes in children.

### 6.1 Interactions during mealtime and non‐mealtime context

With regard to interactions during feeding, our findings show that parents with ED tend to be more intrusive, to display more negative expressed emotions at mealtime, to be involved in higher levels of mealtime conflicts (Stein, 1999; Stein et al., 1994), and to use more verbal statements and controlling, non‐responsive and coercive feeding practices. Stein and colleagues suggested that conflicts were more likely to arise when mothers fail to acknowledge their infant's cues and putting aside their own concerns (Stein, 1999). It is plausible that intense preoccupation with shape and weight demands significant attentional resources, potentially interfering with maternal emotional, cognitive and interpersonal focus on their children (Farrow and Blissett, 2014). However, these findings suggest that while some of the problematic feeding and difficult parent‐child interactions could be a disorder‐specific parenting mechanism, others may be part of the more general impact of parental mental health difficulties on parent‐child relationships. Both of these may contribute to mechanisms through which children may develop difficulties of their own. The extent to which each of these mechanisms impacts the parent‐child relationship is, however, difficult to disentangle. Interestingly, our findings also showed that parents struggling with disordered eating, not necessarily reaching the threshold of criteria for ED, might engage in more coercive and controlling feeding practices, known to be associated with the development of maladaptive eating behaviours in children (Loth et al., 2024). This suggests that even subclinical forms of ED might have an impact on bonding and parent‐child relationship and could potentially contribute to the intergenerational transmission of ED.

Looking at the interactions in a non‐mealtime context, parents with ED seem to experience difficulties more broadly, being more intrusive, less sensitive, and offering less structured guidance compared to controls but similar to those facing other mental health difficulties, like anxiety and depression (Campbell et al., 2004; Tietz et al., 2014). Mothers with ED are more vulnerable to develop comorbidities such as depression and anxiety in the postpartum period compared to controls (Easter et al., 2015), therefore this might be the result of either an independent effect of ED on mother‐child interaction or could reflect the high levels of comorbidity observed in those suffering from ED (Easter et al., 2015). It is also worth noting that ADHD is highly prevalent in people with BN and BED (Villa et al., 2023; Yilmaz et al., 2017). Whilst studies on the impact of comorbid ADHD on bonding are lacking, having a comorbid ADHD could exacerbate challenges like difficulties in maintaining routines, reduced emotional availability and ability to regulate emotions (Johnston et al., 2012; Patel et al., 2002). These factors could affect the parent‐child bond and negatively influence the child's emotional development. Nevertheless, the developmental pathways associated with these mechanisms remain uncertain and require additional research. Future studies are encouraged to disentangle the impact of ED as opposed to comorbid disorders that often co‐occur in those with ED.

Research also shows that people with ED are more likely to have an insecure attachment style and more frequently report that they experienced adverse events such emotional and physical maltreatments compared to general population (Jewell et al., 2023; Micali et al., 2017; Monteleone et al., 2020; Tasca, 2019).

Parents with insecure attachment frequently encounter difficulties in regulating their emotions, struggle with negative self‐image, and may find it challenging to understand and effectively respond to their child's emotional experiences and needs. These difficulties can manifest in inconsistent or emotionally unavailable parenting behaviours, which may hinder the child's ability to form a secure attachment, potentially perpetuating a cycle of insecure attachment across generations (Han et al., 2006; Monteleone et al., 2020).

It has been suggested that insecure attachment could contribute to the development and/or maintenance of an ED (O’Shaughnessy & Dallos, 2009). This could also be an additional risk factor for their children and could potentially reinforce intergenerational transmission of ED and other psychiatric disorders (Han et al., 2006).

Importantly, some of the studies grouped past and current ED together while others used lifetime diagnosis, meaning that their ED might have occurred not concomitantly with the perinatal period. No studies have explored if any differences exist between past and current ED with regards to parent‐child interaction, but one could speculate that the strong food, weight and shape concerns impact the parenting abilities mainly of those with current illness, whilst other mechanisms would be operating in those with past illness such as low self‐esteem, lack of confidence in the ability to be an effective parent. Further research is needed to substantiate this hypothesis.

### 6.2 Bonding, mother‐infant quality of interaction and related outcome in children of mothers with ED

Although the literature is scarce, available evidence suggests that, in the presence of an ED, difficulties in parent‐child interaction may have an impact on children's outcomes. Therefore, we can hypothesise that, in genetically predisposed children, if the ED parent‐child dyad fails to establish mutual attunement and to engage in sensitive interactions, the infant may not be able to acquire the ability of self‐regulation of affect and could exhibit maladaptive behavioural and emotional symptoms as time progresses (Murray et al., 2003). Infants' dissatisfaction with their environment or their relationship with their parents could explain their more difficult temperaments, greater display of internalisation and externalisation problems, more problems relating to their parents (i.e. less responsiveness), and more behavioural difficulties. Additionally, parenting children with externalisation, internalisation, and behavioural difficulties can bring additional challenges, such as emotional strain, social isolation, and educational difficulties, making it particularly challenging for parents already struggling with an ED (Brown et al., 2011).

It is crucial to emphasise that parental ED may heighten the risk of children developing their own mental health challenges, but it does not invariably affect their well‐being and development. Several factors, such as child resilience, availability of mental health care, support networks within families, parenting abilities, and additional parental features, may serve as protective factors for the child. Further research is needed to provide analysis on parent‐child interactions and related child outcomes by historic or active status of diagnoses. This would help to shed some light on the protective factors that mitigate the association between parent‐child interaction and negative child outcomes, ultimately fostering the child's development and healthy adjustment. This would offer a deeper understanding of the mechanisms that are amenable to intervention.

### 6.3 Strengths of evidence

In terms of design, eleven (64.7%) out of 17 were case‐control studies, two (11.8%) had a cohort design and four (23.5%) were uncontrolled. Half of the reviewed studies had sample sizes below 100 participants with two studies (11.8%) having a sample size below 50. This might suggest that the methodological robustness of some of the studies is limited, with some being underpowered. Consequently, it is sensible to interpret the results with caution.

With regard to generalisability, nearly all of the included studies were carried out in Westernised countries, and where demographic information was provided within the manuscripts, participants were predominantly Caucasian. As some ED presentations may vary across different ethnicities (Franko et al., 2007), additional research is necessary to ascertain whether the findings of our review can be applied to more diverse populations.

## 7 LIMITATIONS

This review has several limitations. First, it only incorporates studies published in English, potentially overlooking relevant studies lacking English abstracts, which limits our findings. Also, some of the studies had small sample sizes and lacked a control group.

Additionally, there was considerable heterogeneity across studies in terms of the ED subtypes represented in study samples. Where samples comprised parents with different ED diagnoses, separate analyses by subtype were not always conducted, likely due to small sample sizes. It is possible that mother‐infant quality of interaction and bonding differs across ED subtypes and this should be the scope for future studies. There was significant variation across studies regarding whether parental ED samples included parents with past or current ED, as well as in the methods used to establish ED diagnoses. Ultimately, no studies meeting our inclusion criteria examined parenting in parents with an ED at the stage when their children reach adolescence. This is a surprizing gap, considering that puberty and adolescence are times of heightened vulnerability to developing ED, especially in females (Treasure et al., 2020).

Furthermore, studies differed in terms of whether they considered parents' comorbidities along with ED symptoms, and comorbid symptoms were seldom included in the analysis as a confounding variable. As previously stated, there is a pressing need to disentangle how differences observed in parent‐child interactions between parents with ED and controls may result from comorbid psychopathology as opposed to the impact of ED symptomatology specifically.

### 7.1 Clinical and research implications

Our findings suggest that parents with ED might face difficulties in parent‐child interaction in both feeding and non‐feeding contexts which, in turn, can impact on children's mental health.

Research has proposed that an amendable pathway of risk entails parenting behaviours observed during early interactions between mother and infant (Stein et al., 2014). Innovative parenting interventions aimed at addressing modifiable parenting factors that could impact on the intergenerational transmission of psychiatric disorders have been described and evaluated with positive outcomes for children of depressed (Beardslee et al., 2007) and anxious parents (Cartwright‐Hatton et al., 2018). However, there has been limited exploration of educational programmes designed to assist mothers with ED (Bryant‐Waugh, Turner, Jones, & Gamble, 2007). There is an evident need for accessible and specialised treatment options, that recognise the several difficulties of parenting while managing an ED and offering non‐judgemental support. This support could include professionally facilitated peer support groups, practical strategies for handling feeding and mealtimes, and guidance on navigating interactions with children around ED‐related areas (Chapman et al., 2023).

## 8 CONCLUSIONS AND FUTURE DIRECTIONS

Parents with ED may face difficulties in their interactions with their children, both in feeding and non‐feeding contexts. These difficulties can potentially affect the socio‐emotional functioning and psychopathology of the children. They also suggest that both general and disorder‐specific aspects of parent‐child interaction will be central targets for preventative interventions.

There is a call for more research on fathers with ED, as existing research primarily focuses on mother‐child dyads. Future studies should delve into both feeding and non‐feeding contexts to assess the quality of parent‐child interactions, exploring the influence of specific ED diagnoses and their status (i.e., historical or current) on children's outcomes. Research involving older children is crucial to comprehensively grasp the impact from childhood through adolescence into adulthood. Studies specifically focussing on parental ARFID and its effects on bonding and interactions with children are lacking. This indeed presents an opportunity for future research to explore further this overlooked area. Although not included in our review, to the best of our knowledge, there are no existing studies specifically focused on the bonding between mothers with ED and their children who also have ED. However, this is an important area that presents significant opportunities for future research. Since parental psychopathology may not consistently lead to negative effects on children's well‐being and development, additional research should strive to enhance comprehension of protective factors that moderate the associations between parental ED and detrimental outcomes in children, ultimately fostering the child's healthy adjustment and development. This could provide additional understanding of mechanisms that could be addressed through intervention.

## CONFLICT OF INTEREST STATEMENT

The authors declare no conflict of interest.

## Supporting information

Supporting Information S1

## Data Availability

The data that supports the findings of this study are available in the supplementary material of this article.
