# Supplementary material for: Bonding and parent‐child quality of interaction in parents with eating disorder: A scoping review
Source: Eur Eat Disord Rev. 2024 Oct 18;33(2):221–38. doi: 10.1002/erv.3144 (PMC11786935; doi:10.1002/erv.3144)
Supplement: Supplementary file 1 — Supporting Information S1 [file ERV-33-221-s001.docx]

Appendix 1

| Search terms |
| --- |
| maternal eating disorder* or maternal anorexia or maternal bulimia or maternal binge eating or maternal disordered eating or maternal ARFID* or mother* with eating disorder* or mother* with anorexia or mother* with bulimia or mother* with binge eating or mum* with eating disorder* or mother* with disordered eating or mother* with OSFED or mother* with eating disorder unspecified or mother* with ARFID* or mum* with anorexia or mum* with bulimia or mum* with binge eating or mum* with OSFED or mum* with eating disorder unspecified or mum* with disordered eating or mom* with eating disorder* or mum* with ARFID* or mom* with anorexia or mom* with bulimia or mom* with binge eating or mom* with OSFED or mom* with eating disorder unspecified or mom* with disordered eating or mom* with ARFID or parental eating disorder* or paternal anorexia or paternal bulimia or paternal binge eating or paternal OSFED, or paternal disordered eating or paternal eating disorder unspecified or paternal ARFID or caregiver eating disorder* or postnatal eating disorder* or perinatal eating disorder* or postpartum eating disorder or *parental anorexia or parental bulimia or parental binge eating or parental OSFED, or parental disordered eating or parental eating disorder unspecified or parental ARFID or caregiver eating disorder* or postnatal eating disorder* or perinatal eating disorder* or postpartum eating disorder* |
| AND |
| mother-infant bonding or mother-infant quality of interaction or mother-infant interaction or mother-child bonding or mum-child bonding or mom-child bonding or mum-infant quality of interaction or mum-child quality of interaction or mom-infant quality of interaction or mom-child quality of interaction or parent-infant interaction or parent-child interaction or parent-child relationship quality or mother-child relationship quality or mum-child relationship quality or mom-child relationship quality or mother-child interaction quality or mum-child interaction quality or mom-child interaction quality or parent-child attachment security or mother-child attachment security or mum-child attachment security or mom-child attachment security |
